# Fungal Toxins and Host Immune Responses

**DOI:** 10.3389/fmicb.2021.643639

**Published:** 2021-04-13

**Authors:** Rhys Brown, Emily Priest, Julian R. Naglik, Jonathan P. Richardson

**Affiliations:** Centre for Host-Microbiome Interactions, Faculty of Dentistry, Oral & Craniofacial Sciences, King’s College London, London, United Kingdom

**Keywords:** fungal toxins, mycotoxins, candidalysin, immunity, toxins

## Abstract

Fungi are ubiquitous organisms that thrive in diverse natural environments including soils, plants, animals, and the human body. In response to warmth, humidity, and moisture, certain fungi which grow on crops and harvested foodstuffs can produce mycotoxins; secondary metabolites which when ingested have a deleterious impact on health. Ongoing research indicates that some mycotoxins and, more recently, peptide toxins are also produced during active fungal infection in humans and experimental models. A combination of innate and adaptive immune recognition allows the host to eliminate invading pathogens from the body. However, imbalances in immune homeostasis often facilitate microbial infection. Despite the wide-ranging effects of fungal toxins on health, our understanding of toxin-mediated modulation of immune responses is incomplete. This review will explore the current understanding of fungal toxins and how they contribute to the modulation of host immunity.

## Introduction

Mycotoxins are natural secondary metabolites produced by fungi which grow on a variety of agricultural products including cereals, grains, nuts, spices, apples, dried fruits, and coffee beans (Ukwuru et al., 2017). It is estimated that 20–25% of food crops worldwide contain mycotoxin contamination (Eskola et al., 2020) while mycotoxins and mycotoxin-producing fungi are frequently isolated from construction materials and air samples from water-damaged buildings (Tuomi et al., 2000).

The majority of mycotoxins are synthesised by enzymes present in biosynthetic gene clusters whose first enzyme is a polyketide synthase or a non-ribosomal peptide synthetase (or a mixture of both), with the exception of candidalysin and mucoricin [peptide and protein toxins encoded by *ECE1* and *RLT1* genes, respectively (Gallo et al., 2013; Moyes et al., 2016; Soliman et al., 2021)]. The gut is typically the first point of contact between ingested mycotoxins and the host. The gastrointestinal mucosa (GIM) is a single layer of epithelial cells which are physically connected to one another by desmosomes, tight junctions and adherens junctions. The GIM is colonised by a diverse range of bacteria, archaea, and eukarya which play a critical role in the maintenance of general health. While the protective barrier of the GIM serves to restrict the movement of microbes and toxic substances from the gastrointestinal lumen into the systemic compartment, compromised barrier function and alterations in the composition of the resident microbiota may predispose to disease. The deleterious impact of mycotoxin-induced alterations on intestinal barrier function are reviewed in Gao et al. (2020a).

Mycotoxins can influence the gastrointestinal microbiota directly through antimicrobial activity and through secondary mechanisms involving the release of antimicrobial compounds from mycotoxin-damaged host cells. Numerous studies have investigated the impact of mycotoxins on host microbiota *in vivo* (Guerre, 2020). Conversely, the microbiota can also influence the bioavailability of ingested mycotoxins and their associated metabolites. Mycotoxins can be detoxified through biotransformation (Li et al., 2020) and physically sequestered by direct adsorption onto microbial cell walls which impairs their absorption across the gut. However, it is also worth noting that the process of biotransformation may also generate compounds that are potentially toxic. One notable example of this phenomenon is the bioactivation of “masked” mycotoxins. Plants protect themselves from mycotoxins in a process which typically involves the conversion of mycotoxins into compounds with reduced toxicity (referred to as “masked” mycotoxins) which remain stored in plant tissue. Should plant material containing masked mycotoxins be ingested, host processes and/or the biological activity of the resident microbiota can liberate functional mycotoxins (and related metabolites) which may have detrimental consequences (Berthiller et al., 2013; Gratz, 2017; Gratz et al., 2017).

The predominant routes of exposure to mycotoxins are ingestion and inhalation. However, recent research indicates that some mycotoxins and peptide/protein toxins are associated with fungal infection and contribute to pathogenicity. Fungal diseases are a major cause of morbidity and mortality in the global population and account for an estimated 1.5 million deaths per year (Brown et al., 2012). Compromised immunity and iatrogenic procedures such as gastrointestinal surgery and immunosuppressive therapy are risk factors for the development of life-threatening systemic infections. The saprophytic mould *Aspergillus fumigatus* produces gliotoxin which is detectable in the lungs of patients with invasive aspergillosis (IA; Lewis et al., 2005) while the production of candidalysin by *Candida albicans* contributes to mucosal and systemic disease in a range of experimental models (Naglik et al., 2019).

Innate and adaptive immunity are critical for the control and elimination of infecting microbes. The mucosal barriers of the body, together with immune cells, serve to protect underlying tissues from pathogenic insult and play an active role in microbial recognition and defence. Mucosal surfaces activate and orchestrate cellular responses to microbial infection through the production of cytokines and alarmins which drive the recruitment of specialised innate and adaptive immune cells including macrophages, neutrophils, dendritic cells and T cells to combat invading microbes. However, the majority of mycotoxins have a deleterious effect on the structural integrity of mucosal barriers and are capable of suppressing cellular immunity. Accordingly, toxin-mediated alterations in innate and adaptive host defences may increase susceptibility to infection by pathobionts present in the resident microbiota. This review will explore the impact of mycotoxins, peptide, and protein toxins on the modulation of host immune defence.

## Gliotoxin

**FIGURE 1:**
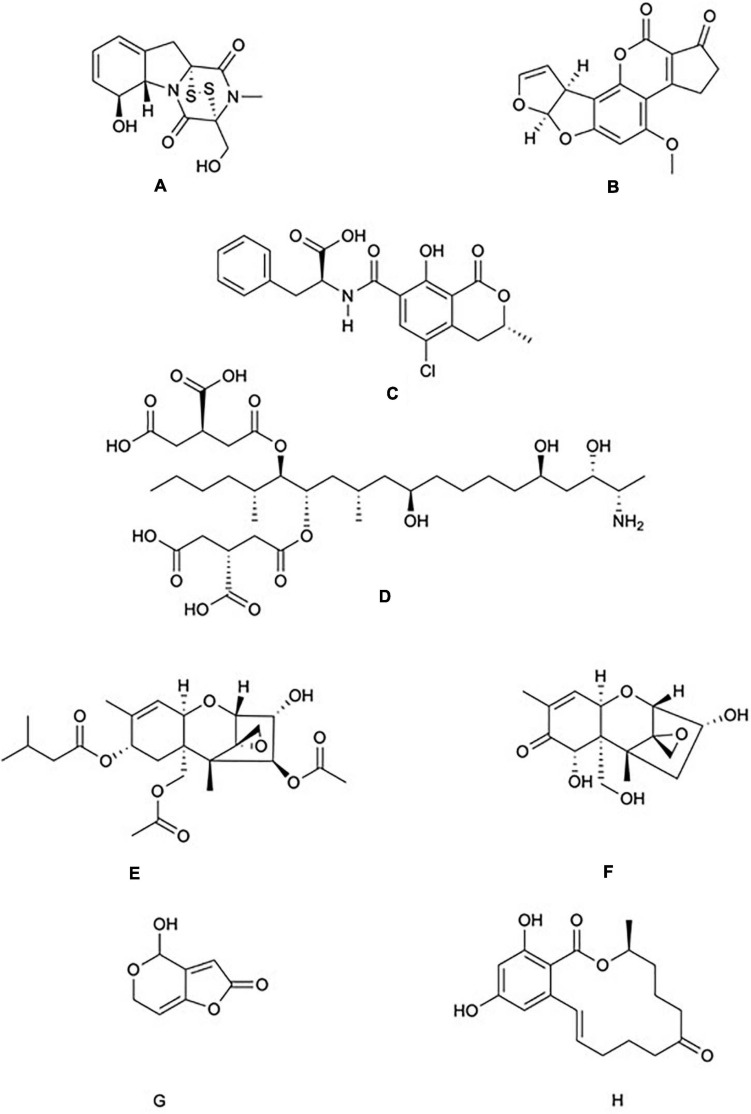
Chemical structure of common mycotoxins. **(A)** Gliotoxin (C_13_H_14_N_2_O_4_S_2_) is a member of the epipolythiodioxopiperazine family of mycotoxins and is produced by *Aspergillus fumigatus*. **(B)** Aflatoxin B1 (C_17_H_12_O_6_) produced by *A. flavus* and *A. parasiticus*. **(C)** Ochratoxin A (C_20_H_18_CINO_6_) is a phenylalanine derivative produced by several species of the genera *Aspergillus* and *Penicillium*. **(D)** Fumonisin B1 (C_34_H_59_NO_15_) is characterised by diester and triol functional groups and is produced by species of the genus *Fusarium*. The trichothecenes family include: **(E)** The type A trichothecene T-2 (C_24_H_34_O_9_) and **(F)** The type B trichothecene Deoxynivalenol (C_15_H_20_O_6_). T-2 and Deoxynivalenol are produced by numerous genera of fungi including *Fusarium*, *Stachybotrys*, and *Trichoderma*. **(G)** Patulin (C_7_H_6_O_4_) is produced by species of the genera *Aspergillus* and *Penicillium*. **(H)** Zearalenone (C_18_H_22_O_5_) is produced by species of the genera *Fusarium* and *Gibberella*.

Gliotoxin ((3*R*,6*S*,10a*R*)-6-hydroxy-3-(hydroxymethyl)-2-methyl −2,3,6,10-tetrahydro-5a*H*-3,10a-epidithiopyrazino[1,2-a]indole -1,4-dione: Figure 1A) is a member of the epipolythiodioxopiperazine (ETP) class of toxins. Numerous studies have suggested that the toxicity of ETP toxins and gliotoxin in particular may be, in part, underpinned by redox cycling of the disulphide bridge which can generate damaging reactive oxygen species (ROS) and drive the formation of mixed disulphide bonds with host proteins (Chai and Waring, 2000). Although gliotoxin was first characterised in the fungal genus *Gliocladium* (Weindling and Emerson, 1936; Weindling, 1941), the fungus most closely associated with gliotoxin is *A. fumigatus*, which causes a range of life-threatening respiratory and systemic infections in patients with compromised immunity. Gliotoxin is detectable in the serum of patients with IA and in the lungs and serum of mice with experimentally induced IA (Lewis et al., 2005) suggesting an association with active infection.

### Epithelial Cells and Gliotoxin

The germination of *A. fumigatus* conidia and subsequent invasion into the respiratory epithelium is a critical step which promotes infection. Gliotoxin promotes cytoskeletal remodelling in human alveolar epithelial cells *in vitro* which facilitates the internalisation of *A. fumigatus* conidia (Jia et al., 2014; Zhang et al., 2019). Fungal burdens present in the lung tissue of immunosuppressed mice infected with an *A. fumigatus gliP*Δ mutant unable to produce gliotoxin were significantly reduced compared with wild-type fungus, and the invasive capacity of the *gliP*Δ mutant was partially restored following the addition of exogenous gliotoxin which correlated with increased mortality (Jia et al., 2014; Zhang et al., 2019). Murine embryonic fibroblasts exposed to 1 μM gliotoxin *in vitro* undergo apoptosis in a Bcl-2 homologous antagonist/killer (Bak) dependent manner (Pardo et al., 2006), while immunosuppressed Bak^–/–^ mice that received an intranasal inoculation of gliotoxin-producing *A. fumigatus* were more resistant to infection compared with wild-type controls, highlighting the role of Bak as critical factor involved in gliotoxin-mediated cell death (Pardo et al., 2006). The application of gliotoxin or exhausted culture medium from wild-type *A. fumigatus* but not a gliotoxin-defective mutant induces apoptosis in human bronchial epithelial cells, and in murine fibroblasts and alveolar epithelial cells in a c-Jun N-terminal kinase (JNK) pathway-dependent manner (Geissler et al., 2013).

### Macrophages and Gliotoxin

Gliotoxin induces apoptosis and cytoskeletal changes that affect macrophage function. Macrophages exposed to 0.3–3 μM gliotoxin *in vitro* undergo DNA fragmentation and apoptosis within 5 h (Waring et al., 1988; Suen et al., 2001). The phagocytic ability of macrophages is significantly diminished through the specific targeting of phosphoinositides. Phosphatidylinositol 3,4,5-trisphosphate (PtdIns(3,4,5)P3) is an important component involved in cytoskeletal remodelling in macrophages during phagocytosis (Levin et al., 2015). Gliotoxin (1.53 μM) induces the dissociation of PtdIns(3,4,5)P3 from primary human and murine RAW 264.7 macrophage plasma membranes which promoted cytoskeletal reorganisation, inhibition of lamellipodia formation, and phagocytosis of zymosan (Schlam et al., 2016).

Cytokines are key molecules in the coordination of immune responses and gliotoxin has been shown to affect cytokine expression and release from macrophages. Gliotoxin (0–4.6 μM) modulates the secretion of interleukin (IL)-6, IL-10, and tumour necrosis factor (TNF)-α from lipopolysaccharide (LPS)-stimulated human MM6 monocytes *in vitro* (Johannessen et al., 2005) while concentrations as low as 107 nM induce apoptosis (Stanzani et al., 2005). Further evidence for the inhibition of cytokine responses is provided by studies in which LPS-stimulated rat macrophages treated with 0.3–3 μM gliotoxin exhibited a reduction in the level of TNF-α secretion *in vitro* (Fitzpatrick et al., 2000).

Recent research has highlighted a critical role for macrophages in toxin surveillance and the maintenance of barrier function in the distal colon. Murine colonocytes are protected from the toxicity of gliotoxin, T-2 toxin and candidalysin by a population of CD11c^high^ subepithelial macrophages which form specialised “balloon-like” protrusions (BLPs) in response to local fungi, and use them to interact with the distal colonic epithelium where they limit the absorption of toxic materials (Chikina et al., 2020). Strikingly, in mice that are depleted for these macrophages, the distal colonic epithelium continues to absorb toxin-containing fluids and undergoes apoptosis concomitant with a loss of barrier integrity. These observations suggest that the BLPs enable colonic epithelial cells to differentiate between harmless and harmful substances and identify CD11c^high^ subepithelial macrophages as central mediators of colonic barrier function and local responses to fungal toxins *in vivo* (Chikina et al., 2020).

### Neutrophils and Gliotoxin

Neutrophils play a major role in the control of fungal infection. Human neutrophils treated with concentrations (92–306 nM) of gliotoxin found in the blood of patients with IA were unable to phagocytose zymosan or serum-opsonised zymosan and exhibited cytoskeletal re-organisation (Coméra et al., 2007). Similarly, isolated polymorphonuclear leukocytes exposed to 107 nM gliotoxin exhibited reduced production of ROS within 30 min (Orciuolo et al., 2007).

The production of neutrophil extracellular traps (NETs) occurs in response to the presence of invading fungi that are too large to be phagocytosed, and the NADPH oxidative burst promotes the formation of NETs in an *in vivo* model of pulmonary aspergillosis (Almyroudis et al., 2013, Röhm et al., 2014). Gliotoxin has been shown to inhibit assembly of the NADPH oxidase in human neutrophils (Tsunawaki et al., 2004).

*In vivo*, immunosuppressed neutropenic mice infected with an *A. fumigatus ΔgliZ* mutant unable to produce gliotoxin exhibited a non-significant improvement in survival after seven days when compared with wild-type controls (Bok et al., 2006). Additionally, a significant increase in the survival of neutropenic mice was observed following disruption of the *A. fumigatus* transcriptional regulator *LaeA*, which was associated with decreased production of gliotoxin and an increased susceptibility to phagocytosis (Bok et al., 2005). No difference in survival was observed between groups of neutropenic mice infected with wild-type *A. fumigatus* conidia or a *ΔgliP* mutant unable to produce gliotoxin (Kupfahl et al., 2006). In contrast, immunosuppressed, non-neutropenic mice infected with the same strains exhibited significantly improved survival (Sugui et al., 2007). Indeed, a *ΔgliP* mutant unable to produce gliotoxin exhibited attenuated virulence in non-neutropenic murine models of invasive pulmonary aspergillosis, but exhibited normal virulence in models rendered neutropenic (Spikes et al., 2008). These observations suggest that non-neutropenic mice are more susceptible to the effects of gliotoxin in a model of IA and highlight neutrophils as a primary target of the toxin.

### Dendritic Cells and Gliotoxin

Dendritic cells are capable of innate recognition of pathogens and also present antigens to naive T cells during adaptive immunity. Gliotoxin (0.1 μM) induced caspase-3 activation and apoptosis in CD83^+^ monocyte-derived dendritic cells while concentrations between 0.15 and 1.5 μM inhibited human T cell function *in vitro* (Stanzani et al., 2005).

### Nuclear Factor kappa-B and Gliotoxin

Nuclear factor kappa-B (NF-κB) signalling plays a role in numerous aspects of innate immunity including immune cell survival and the production of pro-inflammatory M1 macrophages (Ward et al., 1999; Cowburn et al., 2004; François et al., 2005; Wang et al., 2014). Multiple studies have demonstrated that gliotoxin targets NF-κB signalling to promote immune suppression.

**FIGURE 2:**
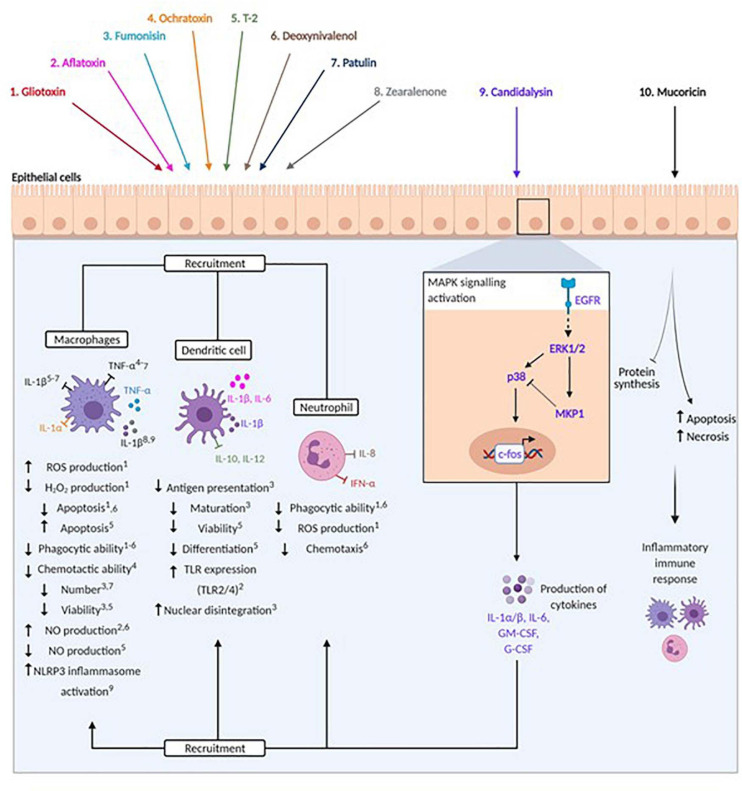
Modulation of host immunity by mycotoxins, peptide and protein toxins. Fungal toxins are represented by colours: 1. Gliotoxin (red), 2. Aflatoxin (pink), 3. Fumonisin (light blue), 4. Ochratoxin (orange), 5. T-2 (green), 6. Deoxynivalenol (brown), 7. Patulin (dark blue), 8. Zearalenone (grey), 9. Candidalysin (purple), 10. Mucoricin (black). Arrows beneath immune cells denote promotion or reduction of indicated processes. Numbers in superscript identify processes affected by specific toxins. Pro- and anti-inflammatory cytokines are indicated by spheres and arrows (stimulation) and blunted arrows (inhibition).

Treatment of Jurkat T cells with 306 nM gliotoxin abolished NF-κB signalling by preventing the degradation of the regulatory subunit IκB-α (Pahl et al., 1996). NF-κB is also implicated in gliotoxin-induced apoptosis of eosinophils. Eosinophils that received TNF-α and 306 nM gliotoxin stabilised IκB-α, resulting in NF-κB inhibition, a significant increase in apoptosis and a decrease in the production of IL-8 (Fujihara et al., 2002). The multiple impacts of gliotoxin on host immune responses are summarised in Figure 2.

## Aflatoxins

Aflatoxins are potent, coumarin ring-containing carcinogens derived from polyketides. There are least 18 different members comprised of aflatoxins and their derivatives (Benkerroum, 2020b) but the most widely studied of these are B1, B2, G1, and G2. Aflatoxin B_1_ ((6a*R*,9a*S*)-2,3,6a,9a-tetrahydro-4-methoxy-1*H*,11*H*-cyclopenta[c]furo[3′,2′:4,5]furo[2,3-h][1]benzopyran-1,11-dione: Figure 1B) was first isolated in 1961 and takes its name from *Aspergillus flavus* (Blount, 1961; Schroeder and Boller, 1973; Saito et al., 1989; Geiser et al., 2000). More recent research has demonstrated that aflatoxins are produced by numerous *Aspergillus* species (Goto et al., 1996; Geiser et al., 2000; Klich et al., 2000; Peterson et al., 2001).

Aflatoxin B_1_ is widely considered to be the most toxic of the aflatoxins. Aflatoxin B_1_ and B_2_ are produced by *A. flavus* and are thus often found as co-contaminants. The toxicity of aflatoxin B_1_ is caused predominantly through the production of the highly unstable intermediate metabolite aflatoxin B_1_ exo-8,9 epoxide (AFBO). AFBO induces cytotoxicity by reacting with host macromolecules including nucleic acids, proteins, and phospholipids (Benkerroum, 2020a). Intriguingly, AFBO is not an intermediate derived from the metabolism of aflatoxin B_2_, hinting at an explanation for the reduced toxicity of aflatoxin B_2_ compared to B_1_ (Benkerroum, 2020a).

Aflatoxins cause a wide range of toxicoses and predominantly target the liver, resulting in cirrhosis and hepatocellular carcinoma (Newberne and Butler, 1969; Shank et al., 1972; Peers, 1973; Mekuria et al., 2020). Indeed, the mean concentration of free aflatoxin B_1_ in the serum of patients with hepatocellular carcinoma was 0.2 nM compared with 0.06 nM in healthy controls (Aydin et al., 2015; Ferri et al., 2017). The presence of aflatoxin-albumin adducts is an often-used biomarker of exposure and adduct concentrations ranging between pg and ng/mL have been detected in African and Asian individuals (Allen et al., 1992; Diallo et al., 1995; Wang et al., 1996; Power et al., 2002). Aflatoxin albumin adducts have also been detected in the bronchoalveolar lavage (BAL) and serum of food-grain workers which correlated with the presence of *Aspergillus* and respiratory symptoms (Malik et al., 2014) while levels ranging between 4.8–12.5 ng/mg and 0.32–0.8 ng/mg were detected in BAL and serum, respectively from patients with chronic lung disease (Ali et al., 2013), suggesting that aflatoxins may be produced by *Aspergillus* during active infection. The presence of aflatoxin B_1_-albumin adducts in the blood plasma of Ghanaian villagers correlated with a significantly lower percentage of activated T cells and lower numbers of activated natural killer cells compared to unexposed individuals (Jiang et al., 2005). Treatment of hepatic cells with 40 μM aflatoxin B_1_ activates the epidermal growth factor receptor (EGFR) which leads to downstream activation of PI3K-AKT/mTOR and induced autophagy (Xu et al., 2020). However, whether aflatoxin-mediated activation of EGFR occurs in immune cells has yet to be determined.

### Epithelial Cells and Aflatoxin

**FIGURE 3:**
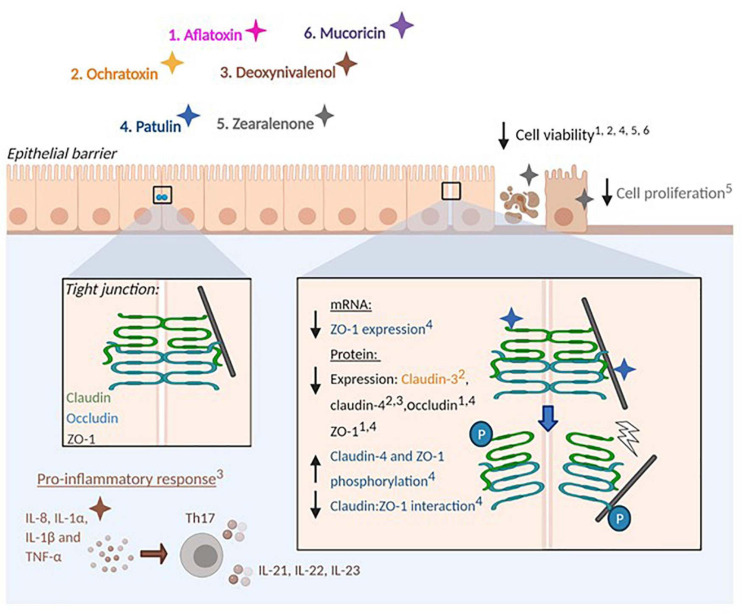
Effect of mycotoxins and protein toxins on barrier integrity. Fungal toxins that influence epithelial barrier integrity are represented by colours: 1. Aflatoxin (pink), 2. Ochratoxin (orange), 3. Deoxynivalenol (brown), 4. Patulin (blue), 5. Zearalenone (grey). 6. Mucoricin (purple). Mycotoxins impair epithelial barrier integrity by altering the production of tight junction proteins (Claudins, Occludin and Zonula occludens-1) by epithelial cells. Arrows denote the promotion or reduction of indicated processes. Numbers in superscript identify processes affected by specific toxins. Protein phosphorylation is indicated (P). Mycotoxins also stimulate pro-inflammatory responses at mucosal surfaces and impair barrier integrity in a tight junction-independent manner by reducing cell viability and proliferation.

Aflatoxins have a significant effect on the integrity of epithelial barriers. Aflatoxin M_1_ is a hydroxylated metabolite of aflatoxin B_1_ which is detectable in a variety of dairy products (Bahrami et al., 2016). Differentiated gastrointestinal epithelial cells exhibit significant reductions in trans-epithelial electrical resistance (TEER) following exposure to aflatoxin B_1_ (100 μM) and M_1_ (0.12–12 μM) which correlated with a reduction in the expression of tight junction proteins and decreased viability (Romero et al., 2016; Gao et al., 2017). The effects of aflatoxin (and other fungal toxins) on barrier integrity are summarised in Figure 3.

### Monocytes, Macrophages, and Aflatoxin

Human monocytes exposed to aflatoxin B_1_ exhibit a significant reduction in the ability to phagocytose and kill *Candida albicans in vitro* (Cusumano et al., 1996), while aflatoxin M_1_ (25 and 50 μg/kg body weight) administered to mice over a 28 day period caused a reduction in the phagocytosis of *Escherichia coli* by monocytes (Shirani et al., 2018).

The production of nitric oxide (NO) by immune cells inhibits the growth of invading microbes and regulates inflammatory innate immune responses (Bogdan et al., 2000). CD14-mediated recognition of LPS by macrophages drives the production of NO which induces cytotoxic effects in numerous pathogens. Application of aflatoxin B_1_ (0–50 μM) to murine peritoneal macrophages prior to LPS stimulation suppressed the expression of CD14 and reduced the production of NO (Moon and Pyo, 2000). Aflatoxin B_1_, B_2_ and their metabolic products aflatoxin M_1_ and M_2_ have similar effects on NO production. Treatment of murine macrophages with 30 μM of these molecules caused a modest but significant (∼5–40%) reduction in NO production.

Interestingly, when macrophages are exposed to a combination of 15 μM aflatoxin B_1_ and M_1_, or aflatoxin B_2_ and M_2_, a synergistic reduction in NO production occurs which was more pronounced when compared to aflatoxin M_1_ or M_2_ alone (Bianco et al., 2012). These observations suggest that aflatoxins and their metabolic products may work co-operatively to suppress macrophage function.

Aflatoxin B_1_ does not promote macrophage apoptosis but rather induces cell cycle arrest. J774A.1 murine macrophages treated with 50 μM aflatoxin B_1_ for 24 h induced a significant switch from G0/G1 phase to S phase (Bianco et al., 2012). While apoptosis is not induced by aflatoxin B_1_, an autophagic response and release of macrophage extracellular traps (METs) was observed in a ROS-dependent manner (An et al., 2017). Finally, aflatoxin B_1_, B_2_, and G_2_ supressed the ability of primary rat peritoneal macrophages to phagocytose and kill *C. albicans in vitro* (Cusumano et al., 1990).

### Neutrophils and Aflatoxin

Human neutrophils treated with 32 nM aflatoxin B_1_ exhibited a depletion of intracellular ATP and a non-significant increase in apoptosis (Mehrzad et al., 2020), while treatment of polymorphonuclear leukocytes with 160 nM aflatoxin B_1_ induced a significant reduction in ROS production and chemotactic ability compared to controls (Ubagai et al., 2008). Studies also demonstrate a reduction in the chemotactic responses of neutrophils isolated from the blood of piglets, and a reduction in phagocytosis and intracellular killing of *Staphylococcus aureus* and *E. coli* in neutrophils isolated from dairy cows (Silvotti et al., 1997; Mehrzad et al., 2011).

### Dendritic Cells and Aflatoxin

Human monocyte-derived dendritic cells treated with 32 nM aflatoxin B_1_ upregulate the expression of toll-like receptor (TLR) 2/4 and the secretion of IL-1β and IL-6 within 2 h (Mohammadi et al., 2014). In contrast, porcine monocyte-derived dendritic cells treated with 32 nM aflatoxin B_1_ for 12 h exhibited impaired phagocytosis and reduced expression of co-stimulatory CD40 although the secretion of IL-1β, IL-8, IL-10, and TNF-α remained unchanged. After 24 h of exposure an impairment in the ability to induce T cell proliferation was observed (Mehrzad et al., 2014). The effects of aflatoxin on host immune responses are summarised in Figure 2.

## Ochratoxin

The ochratoxins contain a 3,4-dihydro-3-methylisocoumarin moiety coupled to β-phenylalanine. While three ochratoxins are known to exist (A, B, and C), ochratoxin A (*N*-{[(3*R*)-5-chloro-8-hydroxy-3-methyl-1-oxo-3,4-dihydro-1*H*-isochromen-7-yl]carbonyl}-Lphenylalanine: Figure 1C) is the most prevalent. Ochratoxin A was discovered in *Aspergillus ochraceus* in 1965 (van der Merwe et al., 1965) and is produced by numerous *Aspergillus* and *Penicillium* species (Abarca et al., 1994; Bayman et al., 2002; Cabañes et al., 2010). Ochratoxins are associated with nephrotoxicity in humans and in animal models (Boorman et al., 1992; Betbeder et al., 1995; Maaroufi et al., 1995) and exert broad immunotoxicity on the mammalian immune system (Thurston et al., 1986; Richard, 1991). Exposure of piglets to sub-chronic levels (0.05 mg/kg in feed) of ochratoxin A for 30 days caused a reduction in NF-κB gene expression in duodenum and in the expression of several cytokines including IL-8, IL-17A, and IL-18 in duodenum and colon (Marin et al., 2017).

### Epithelial Barriers and Ochratoxin

Ochratoxin A has a significant effect on the integrity of the gastrointestinal barrier. Caco-2 monolayers treated with 100 μM ochratoxin A exhibited a time-dependent reduction in TEER between 4 and 24 h which correlated with an absence of claudin-2 (McLaughlin et al., 2004). Similarly, application of 2, 4, and 8 μM ochratoxin A to porcine intestinal epithelial cells stimulated apoptosis in a dose-dependent manner (Wang et al., 2017).

### Macrophages and Ochratoxin

Mice that received intraperitoneal administration (3 and 6 mg/kg body weight per day) of ochratoxin A for up to 15 days produced monocytes with an impaired ability to phagocytose *E. coli* (Müller et al., 1995), while daily oral administration (1.5 mg/kg body weight) over 17 weeks impaired the chemotactic activity of peritoneal macrophages and production of IL-1 and TNF-α (Dhuley, 1997).

Bovine macrophages treated with 9 μM ochratoxin A for 6 and 24 h differentially expressed genes involved in oxidative stress responses and apoptosis (Brennan et al., 2017). In contrast however, a recent *in vitro* study conducted on porcine alveolar macrophages revealed a temporal, biphasic response to 2.5 μM ochratoxin A. At 24 h post-treatment, expression of IL-6 and TNF-α was significantly upregulated and the majority of macrophages were pro-inflammatory CD80^+^ M1 cells. By 72 h post-treatment, macrophage viability was significantly reduced, IL-6 and TNF-α expression returned to baseline levels and a significant switch in macrophage polarisation to a CD208^+^ anti-inflammatory M2 phenotype was observed (Su et al., 2019).

### Neutrophils and Ochratoxin

Mice that received a single oral dose (10 mg/kg body weight) of ochratoxin A exhibited increased oxidative damage of parenchymal organs and neutrophil infiltration into the duodenum after 24 h (Ferrante et al., 2006). Notably however, the phagocytic ability of neutrophils was significantly reduced after 17 days (Müller et al., 1995), suggesting that cellular function was impaired. Application of ochratoxin A to human neutrophils *in vitro* stimulated a dose-dependent activation of the oxidative burst response from 2 h, an increase in the level of intracellular calcium from 4 h and adenosine triphosphate depletion and necrosis from 8 and 20 h, respectively (Kupski et al., 2016), while 25 μM treatment impaired chemotactic and NO responses (Richetti et al., 2005).

### Inflammatory Disease and Ochratoxin

Despite evidence showing that ochratoxins have an immuno-suppressive effect, pro-inflammatory responses were observed following gastric intubation of 49.5 nM ochratoxin A which promoted the progression of rheumatoid arthritis (RA) *in vivo* (Jahreis et al., 2017). An increase in the prevalence and clinical severity of RA was observed in a collagen-induced murine model which correlated with increased production of IL-1β and IL-6 in inflamed joints and IFN-γ and IL-17 in splenocytes. Furthermore, increased production of IL-1β, IL-6, and TNF-α from activated murine macrophages was observed in response to 2.5–250 nM ochratoxin A and facilitated the differentiation of naïve T cells into Th1 cells *in vitro* (Jahreis et al., 2017). The modulatory influence of ochratoxins on host immune responses are summarised in Figure 2.

## Fumonisin

The fumonisins are polyketides which are structurally similar to sphingosine. Fumonisin B_1_ ((2*S*,2′*S*)-2,2′-{[(5*S*,6*R*,7*R*,9*R*,11*S*,16*R*,18*S*,19*S*)-19-Amino-11, 16, 18-trihydroxy-5,9-dimethylicosane-6,7-diyl]bis[oxy(2-oxoethane-2, 1-diyl)]}disuccinic acid: Figure 1D) was discovered in 1988 and is produced by numerous species of *Fusarium* including *Fusarium oxysporum*. Fumonisins and fumonisin-producing fungi have broad immunotoxic effects on livestock *in vitro* and *in vivo* including reduced number, viability and phagocytic ability of macrophages, blockade of dendritic cell maturation and antigen presentation, diminished antigen specific T cell responses and a reduction in the size of spleen and thymus (Marijanovic et al., 1991; Qureshi and Hagler, 1992; Chatterjee et al., 1995; Qureshi et al., 1995; Pierron et al., 2016).

### Macrophages and Fumonisin

Fumonisins exert a broad inhibitory effect on the function of livestock macrophages. Swine alveolar macrophages exposed to different concentrations of fumonisin B_1_ exhibited DNA laddering (7 and 35 μM) consistent with apoptosis, impaired phagocytosis (69 nM and 0.7 μM) and a reduction in the level of IL-1β and TNF-α gene expression (2.7 and 14 μM) *in vitro* (Liu et al., 2002). Similarly, a reduction in IL-1β, IL-6, and IL-8 gene expression was observed in the spleen of piglets that received dietary (6 mg/kg) fumonisin B_1_ over 5 weeks (Grenier et al., 2011). Chicken peritoneal macrophages exhibited reduced phagocytic capacity, cytoplasmic blebbing, nuclear disintegration, and significant cell death in response to fumonisin B_1_ *in vitro* (Qureshi and Hagler, 1992).

### Dendritic Cells and Fumonisin

Piglets that received an oral dose of fumosinin B_1_ (1 mg/kg body weight) for 10 days and were subsequently exposed to purified F-4 fimbriae from enterotoxigenic *E. coli* exhibited prolonged intestinal infection, reduced intestinal expression of IL-12p40 and an impairment in the function of intestinal antigen presenting cells. Moreover, dendritic cells treated with fumosinin B_1_ were impaired in their ability to activate T cells *in vitro* (Devriendt et al., 2010). Collectively, these data suggest that Fumonisin B_1_ dampens the immune response to pathogenic enteric bacteria through the modulation of immune cell activity (Devriendt et al., 2010). The impact of fumonisins on host immune responses are summarised in Figure 2.

## T-2 Toxin

T-2 toxin (Figure 1E) is a type A member of the trichothecene family of mycotoxins produced by many of the same fungi that also produce deoxynivalenol (*Fusarium, Isaria, Microcyclospora, Myrothecium, Peltaster, Spicellum, Stachybotrys, Trichoderma*, and *Trichothecium*) (Venkatasubbaiah et al., 1995; Kikuchi et al., 2004; McCormick et al., 2011; Surup et al., 2014). T-2 toxin is a potent inhibitor of protein synthesis and is associated with conditions including alimentary toxic aleukia and Kashin-Beck disease (Bony et al., 2007; Wan et al., 2015; Li et al., 2016).

### Macrophages and T-2 Toxin

Porcine alveolar macrophages treated with 30 nM T-2 toxin for 16 h exhibited a significant increase in apoptosis concomitant with decreased mitochondrial membrane potential (Seeboth et al., 2012). Moreover, pre-treatment of the same cells with 3 nM T-2 toxin for 1 h followed by exposure to TLR 2, 4, and 2/6 agonists for 16 h induced a significant reduction in TLR2, TLR4, and TLR2/6 mRNA expression, and reduced production of IL-1β and NO compared with non-pre-treated controls (Seeboth et al., 2012).

Treatment of human macrophages with 10 nM T-2 toxin significantly reduced the oxidative burst response and phagocytosis of fluorescent microspheres, while pre-treatment with 10 nM T-2 toxin followed by LPS stimulation caused a significant reduction in the secretion of TNF-α compared with non-pre-treated controls (Hymery et al., 2009). These observations suggest that T-2 toxin may diminish the hosts ability to recognise and respond to pathogens which may increase susceptibility to infection.

### Dendritic Cells, Monocytes, and T-2 Toxin

Dendritic cells are critical conduits that link innate and adaptive immunity. Treatment of immature human monocyte-derived dendritic cells with 1 μM T-2 toxin *in vitro* reduced viability by 90% after 24 h, while dendritic cells pre-treated with LPS and 10 nM T-2 toxin exhibited a marked reduction in the expression of the dendritic cell maturation marker CD86 and reduced secretion of IL-10 and IL-12 when compared with LPS stimulation alone (Hymery et al., 2006).

Human primary monocytes are sensitive to significantly lower concentrations of T-2 toxin than differentiated macrophages or dendritic cells. Monocytes treated with 0.1 nM T-2 toxin for 24 h exhibited a significant decrease in viability compared to controls, while approximately 10-fold higher concentrations were required to significantly reduce the viability of macrophages and dendritic cells (Hymery et al., 2006, 2009; Seeboth et al., 2012). As well as cytotoxic effects, cellular differentiation is also negatively affected by T-2 toxin. Human primary monocytes cultured with T-2 toxin (0.1–10 nM) and granulocyte macrophage-colony stimulating factor (GM-CSF) significantly reduced the expression of CD71 (a marker of macrophage differentiation) compared to cells cultured with GM-CSF alone (Hymery et al., 2009). The differentiation of monocytes into dendritic cells can be monitored by quantification of CD14 (monocyte marker) and CD1a (dendritic cell marker). Human primary monocytes cultured with GM-CSF and IL-4 were 85% CD1a^+^ after 48 h, while 80% of monocytes similarly cultured in the presence of 10 nM T-2 toxin remained CD14^+^ (Hymery et al., 2009). The impact of T-2 toxin on host immune responses is summarised in Figure 2.

## Deoxynivalenol

Deoxynivalenol ((3α,7α)-3,7,15-trihydroxy-12,13-epoxytrichothe -9-en-8-one: Figure 1F) is a type B member of the trichothecene family of mycotoxins and has been isolated from numerous genera of fungi including *Fusarium, Isaria, Microcyclospora, Myrothecium, Peltaster, Spicellum, Stachybotrys, Trichoderma*, and *Trichothecium* (Venkatasubbaiah et al., 1995; Kikuchi et al., 2004; McCormick et al., 2011; Surup et al., 2014). Like T-2 toxin, deoxynivalenol is also a potent inhibitor of eukaryotic protein synthesis and although it is considered to be a less potent toxin than T-2, it exerts significant toxicity on humans and animals and has the greater effect on innate immunity. Acute symptoms following ingestion include: diarrhoea, vomiting, nausea, and gastroenteritis earning deoxynivalenol the name of “vomitoxin” (Forsyth et al., 1977; Friend et al., 1982; Prelusky and Trenholm, 1993; Luo, 1994; Iverson et al., 1995).

### Gut Epithelium, Inflammatory Bowel Disease, and Deoxynivalenol

Like many mycotoxins, deoxynivalenol causes disruption to intestinal barriers. Deoxynivalenol-induced activation of p44/42 ERK signalling inhibited the expression of claudin-4 in IPEC-1 intestinal epithelial cells, resulting in the loss of gastrointestinal barrier integrity (Pinton et al., 2010). In addition to perturbation of the gut mucosal barrier deoxynivalenol also induces phenotypes associated with inflammatory bowel disease *in vitro* and *in vivo.* IPEC-1 cells treated with 10 μM deoxynivalenol induced significant expression of the pro-inflammatory cytokines IL-8, IL-1α, IL-1β, and TNF-α by 4 h which correlated with the activation of genes associated with a pathogenic, pro-inflammatory Th17 response (IL-21, IL-22, IL-23) (Cano et al., 2013). Furthermore, a rat model of DSS-induced colitis exposed to 8 mg/kg of deoxynivalenol in their diet for 4 weeks induced a more rapid and severe onset of colitis than controls and induced significant increases in pro-inflammatory markers (myeloperoxidase, CXCL-1, and IL-1β) (Payros et al., 2020). These observations suggest that deoxynivalenol may be a risk factor in the onset of inflammatory bowel disease and may involve Th17 responses.

### Macrophages and Deoxynivalenol

Though the trichothecenes are potent inhibitors of protein synthesis, deoxynivalenol is capable of stabilising mRNA transcripts that encode pro-inflammatory cytokines through the interaction of HuR/ELAVL1 RNA binding protein with the 3′ untranslated region of mRNA (Wong et al., 2001; Choi et al., 2009). Indeed, treatment of LPS-stimulated RAW276.4 macrophages with 0.84 μM deoxynivalenol for 6 h upregulated the expression of IL-1β, IL-6, and TNF-α (He et al., 2013).

Cyclooxygenase-2 (COX-2) is a rate limiting enzyme that catalyses the production of prostaglandins and thromboxane A_2_, both of which play a key role in the inflammatory response (Vane et al., 1998; Smith et al., 2000). Expression of COX-2, IL-8, and TNF-α mRNA in response to deoxynivalenol is driven by ERK and p38 signalling, and in part mediates the expression of IL-6 and IL-1β with mRNA stability also promoted by p38 (Moon and Pestka, 2002; Chung et al., 2003; Islam et al., 2006). As well as the induction of pro-inflammatory responses, deoxynivalenol also induces caspase-3 activation in a dose-dependent manner in J7741.A murine macrophages, suggestive of apoptosis (Marzocco et al., 2009). Independent inhibition of ERK and p38 signalling confirmed that these factors also play a role in apoptotic cell death induced by deoxynivalenol (Yang et al., 2000; Marzocco et al., 2009).

### Neutrophils, *in vivo* Models, and Deoxynivalenol

Deoxynivalenol reduces chemotaxis, IL-8 secretion and phagocytic functionality of LPS-stimulated porcine neutrophils which correlated with p38 phosphorylation, caspase-3 activation and apoptosis (Gauthier et al., 2013). Mice that received an oral gavage of deoxynivalenol (5–25 mg/kg body weight) robustly induced the expression of genes encoding IL-1β, IL-6, TNF-α, and Th_1/2_ cytokines in spleen tissue 2 h post treatment (Zhou et al., 1997). A single oral dose (12.5 mg/kg body weight) of deoxynivalenol administered to mice induced significant production of IL-1β, IL-6, and TNF-α mRNA at 3-6 h which correlated with JNK, ERK1/2, and p38 phosphorylation (Zhou et al., 2003).

### NF-κB and Deoxynivalenol

Nuclear factor kappa-B is a target of deoxynivalenol, however, there are conflicting reports regarding its effect. Mice given a single oral dose of deoxynivalenol (25 mg/kg body weight) induced nuclear translocation of p50 and c-Rel NF-κB subunits in spleen tissue (Zhou et al., 2003). Conversely, RAW264.7 macrophages treated with 0.2–3.3 μM deoxynivalenol and stimulated with LPS for 6 h exhibited significant reductions in NF-κB luciferase reporter activity while higher concentrations 1.7–3.3 μM inhibited IκBα phosphorylation which correlated with MyD88 inhibition (Sugiyama et al., 2016). The effects of deoxynivalenol on host immune responses are summarised in Figure 2.

## Patulin

Patulin (4-hydroxy-4*H*-furo[3,2-c]pyran-2(6*H*)-one: Figure 1G) is a polyketide most commonly produced by the fungal genera *Penicillium* and *Aspergillus* (Frisvad, 2018). Patulin is produced in a 10-step biosynthetic pathway that is controlled by 15 genes (Puel et al., 2010; Li et al., 2019). Mice that received an intraperitoneal injection of patulin (1 mg/kg body weight) exhibited a significant increase in the level of ROS in hepatic tissue (Song et al., 2014; Guerra-Moreno and Hanna, 2017; Sun et al., 2018) while 2 μM patulin induced autophagy in HepG2 liver cells *in vitro* (Sun et al., 2018), suggesting that patulin may have a detrimental impact on liver function in addition to immunotoxicity.

### Epithelial Cells and Patulin

Intestinal barriers are a major target of patulin. Caco-2 intestinal epithelial cells exposed to 50 μM patulin for 1-24 h exhibited reductions in TEER, cell viability and reduced expression of zonula occludens-1 (ZO-1) and myosin light chain-2 (MLC2), but not occludin, claudin-1 or claudin-3 (Assunção et al., 2016). Similar treatment conditions induced a reduction in ZO-1 and claudin-4 protein at cell-cell junctions after 24 h, while the interaction between ZO-1 and claudin-4 was abrogated after 72 h (Kawauchiya et al., 2011), suggesting that barrier integrity is negatively affected by patulin.

Caco-2 cells treated with 50 μM patulin for 6 h exhibited a marked reduction in the expression of density-enhanced phosphatase-1 (DEP-1), and the peroxisome proliferator-activated receptor gamma (PPARγ) transcription factor (Katsuyama et al., 2014). Knockdown of *DEP-1* expression stimulated claudin-4 phosphorylation, a reduction in ZO-1 and the degradation of epithelial tight junctions (Katsuyama et al., 2014), suggesting that DEP-1 and PPARγ may be targeted by patulin to reduce gastrointestinal barrier function.

### Macrophages and Patulin

Bovine macrophages reduced the expression of genes encoding IL-23, IL-10 and TGF-β in response to 0.3 μM patulin treatment and exhibited impaired ability to phagocytose mCherry-labelled *Mycobacterium avium* ssp. *Paratuberculosis* (Oh et al., 2015). Murine peritoneal macrophages exposed to 0.65–13 μM patulin for 2 h also displayed an impairment in phagocytosis of *Saccharomyces cerevisiae*, reduced production of lysozyme and fungal killing (Bourdiol et al., 1990). Moreover, rats that received 0.1 mg/kg (body weight) of patulin per day displayed mitochondrial abnormalities after 60 days and an increase in the production of apoptotic bodies after 90 days (Özsoy et al., 2008). LPS-stimulated J774A.1 murine macrophages treated with 0.0065–6.5 pM patulin exhibited reduced secretion of IL-6 and TNF-α *in vitro* (Loftus et al., 2016), while LPS-dependent production of IL-6 and NO was blocked in RAW264.7 macrophages in response to 5–100 μM patulin and cell viability was markedly reduced at concentrations above 50 μM *in vitro* (Tsai et al., 2016). Furthermore, the induction of NOD-, LRR-, and pyrin domain-containing protein 3 (NLRP3) and pro-IL-1β in LPS-primed J774.1 macrophages was reduced by patulin, suggesting that inflammasome responses are also negatively affected (Tsai et al., 2016).

### Glutathione and Innate Immune Suppression by Patulin

Patulin-induced modulation of intracellular glutathione (GSH) plays a major role in immunosuppression. Patulin depletes intracellular pools of GSH in CD3 and CD28 antibody-activated human peripheral blood mononuclear cells which correlated with a reduction in cytokine secretion. Indeed, treatment with 81 nM patulin was sufficient to reduce secretion of IFN-γ and IL-10, while a higher concentration (0.65 μM) was required to reduce the secretion of IL-4 and IL-13 (Luft et al., 2008). TLR ligand-induced secretion of IL-6 from murine RAW264.7 and primary peritoneal macrophages was blocked by pre-treatment with 1 μM patulin and rescued by the addition of GSH (Tsai et al., 2016).

The immunotoxicity of patulin is in part due to the induction of mitochondrial dysfunction which is dependent upon GSH depletion. The application of 1 μM patulin to rat hepatocytes was sufficient to induce rapid depletion of GSH (within 20 min), while 1 mM patulin induced the production of ROS, depolarisation of the mitochondrial membrane, an increase in the level of intracellular calcium and intracellular acidification (Barhoumi and Burghardt, 1996). Similarly, 1 μM patulin induced mitochondrial translocation of p62 (autophagy marker) and mitophagy in murine RAW264.7 macrophages (Tsai et al., 2016). The suppressive effects of patulin on host immune responses are summarised in Figure 2.

## Zearalenone

Zearalenone ((3*S*,11*E*)-14,16-dihydroxy-3-methyl-3,4,5,6,9,10-hexahydro-1*H*-2-benzoxacyclotetradecine-1,7(8*H*)-dione: Figure 1H) is produced by numerous species of *Fusarium* fungi (Hestbjerg et al., 2002; Kim et al., 2005; EFSA, 2013). Zearalenone and its metabolic products are non-steroidal oestrogenic mycotoxins that bind to oestrogen receptors, thereby influencing the proliferation of oestrogen-dependent cells *in vitro*. Oestrogen-dependent MCF-7 breast cancer cells treated with zearalenone or its derivatives α-zearalenol or β-zearalenol at concentrations between 6.25 and 12.5 μM undergo significant cellular proliferation compared with untreated controls (Tatay et al., 2018). In addition to this agonist response, zearalenone also exerts broad-ranging toxicity on host cells through a variety of mechanisms discussed below.

### Epithelial Cells, Macrophages, and Zearalenone

*In vitro* studies of kidney and colonic epithelial cells demonstrate that concentrations of zearalenone between 1 and 60 μM are sufficient to reduce cellular proliferation, inhibit DNA, RNA and protein synthesis by 50% and induce lipid peroxidation (Ghédira-Chékir et al., 1999; Abid-Essefi et al., 2004; Kouadio et al., 2005). The production of apoptotic bodies occurs at similar concentrations (Abid-Essefi et al., 2003), suggesting that programmed cell death is induced.

Porcine jejunum enterocytes treated with 25 μM zearalenone for 24 h upregulated the production of caspase-1, pro-IL-1β, and pro-IL-18 mRNA transcripts while an increase in ROS production and reduction in mitochondrial membrane potential and cell viability was observed after 48 h (Fan et al., 2017, 2018).

Mice that received an oral gavage of zearalenone (20 mg/kg body weight) once daily for one week displayed alterations in the structure of the intestinal mucosa while the expression of β-defensin, mucin-1, mucin-2, IL-1β, TNF-α, and secretory immunoglobulin A were significantly increased (Wang et al., 2018). These observations suggest that zearalenone reduces the viability of cells involved in the maintenance of intestinal barrier function while stimulating a pro-inflammatory response. Mice given 4.5 mg/kg bodyweight of zearalenone for 1 week expressed IL-1β, IL-18, and MPO mRNA transcripts and NLRP3 protein in colonic tissue and exhibited colitis-like symptoms (Fan et al., 2018).

In contrast to epithelial cells, relatively little is known about the biological activity of zearalenone on macrophages. However, murine RAW 264.7 macrophages stimulated with LPS for 16 h were observed to upregulate the expression of genes encoding IL-1β, IL-6, and TNF-α in response to 100 nM zearalenone *in vitro* (Pestka and Zhou, 2006).

### Immunotoxicity of Zearalenone Metabolites

Once ingested, zearalenone can be metabolised into several different compounds, each with differing levels of immunotoxicity. Although biotransformation of zearalenone occurs predominantly in the liver, human intestinal epithelial cells can also metabolise zearalenone into α-zearalenol and β-zearalenol *in vitro* (Videmann et al., 2008) and both of these metabolites are detectable in human urine after 24 h following oral administration of zearalenone (Mirocha et al., 1981). Both α-zearalenol and β-zearalenol caused a reduction in IL-8 secretion and cell viability in primary porcine neutrophils that was more potent than that induced by zearalenone (Marin et al., 2010), suggesting that the products of zearalenone metabolism may exert a greater influence on host immunity. The immunomodulatory effects of zearalenone are summarised in Figure 2.

## Combinatorial Exposure to Mycotoxins

Multiple mycotoxins can be produced from a single species of fungi, and it is not uncommon for foodstuffs to be simultaneously contaminated with multiple species of mycotoxin-producing fungi. Indeed, combinations of mycotoxins are detectable in contaminated food (Alshannaq and Yu, 2017; Jin et al., 2021; Xiong et al., 2021). Accordingly, it is feasible that the ingestion of contaminated foodstuff may result in exposure to multiple toxins which may exacerbate immunotoxicity.

Intestinal epithelial cells respond to a combination of aflatoxin M_1_ and ochratoxin A (12 and 10 μM, respectively) by upregulating the expression of genes encoding IL-1α, IL-1β, IL-6, TNF-α, and IFN-α when compared with aflatoxin M_1_ alone (Gao et al., 2020b), while a combination of the same mycotoxins (12 and 20 μM, respectively) reduces the expression of claudin-3, claudin-4, occludin and ZO-1 when compared to individual treatment (Gao et al., 2017). In contrast, porcine intestinal enterocytes treated for 48 h with a combination of deoxynivalenol and fumonisin B_1_ (0.5 and 20 μM, respectively), zearalenone and fumonisin B_1_ (10 and 20 μM, respectively), or a mixture comprising all three toxins exhibited reduced cellular viability *in vitro*. Importantly, the concentration of each individual toxin was non-cytotoxic, suggesting that exposure to mycotoxins at seemingly non-toxic concentrations can exert toxicity when combined (Wan et al., 2013).

Swine alveolar macrophages treated with a combination of ochratoxin A and aflatoxin B_1_ (1 and 0.5 μM, respectively) for 48 h displayed a reduction in the level of intracellular GSH and phagocytic index (Hou et al., 2018).

Bovine macrophages treated with a combination of ochratoxin A (8.9 μM) and patulin (0.32 μM) downregulated the expression of genes encoding DNA methyltransferase 3a (*DNMT-3a*) and histone deacetylase 3 (*HDAC-3*), suggesting that combinatorial exposure may modulate epigenetic processes (Oh et al., 2013).

## Peptide Toxins: Candidalysin

*Candida albicans* is a common commensal of the human microbiota which can become pathogenic when host immunity is compromised. Candidalysin (SIIGIIMGILGNIPQVIQIIMSIVKAFKGNK) is a peptide toxin generated from its parent protein (Ece1p) via enzymatic processing by fungal kexins and secreted from the invasive hyphal morphology of *C. albicans* (Moyes et al., 2016; Richardson et al., 2018a; Naglik et al., 2019). Candidalysin is amphipathic, adopts an α-helical structure, and permeabilises artificial membranes through pore-formation, thus damaging host cells (Moyes et al., 2016).

### Epithelial Cells and Candidalysin

In oral and vaginal epithelial cells, 15–70 μM candidalysin induces the release of lactate dehydrogenase and other alarmins (Moyes et al., 2016; Richardson et al., 2018b; Ho et al., 2020), which are characteristics of cell damage and membrane destabilisation. Candidalysin (1.5–70 μM) activates epithelial immunity via two distinct mitogen-activated protein kinase (MAPK) signalling pathways; p38 and ERK1/2 (Moyes et al., 2010, 2016), with ERK1/2 being driven by activation of the EGFR (Ho et al., 2019). This leads to cytokine release, particularly G-CSF, GM-CSF, and IL-6, which is predominantly directed by the activator protein-1 (AP-1) transcription factor c-Fos and regulated by the MAPK phosphatase MKP1 (Ho et al., 2019). MAPK-dependent release of cytokines and damage-induced release of IL-1 family members (e.g., IL-1α/β) induces downstream innate immune cellular responses, including neutrophil recruitment and innate Type-17 immunity (comprising γδ-T cells and “natural” CD4^+^TCRαβ^+^ T cells). These cells are critical for protection against mucosal candidiasis in both murine models and in human antifungal immunity (Verma et al., 2017). Notably, immune responses against *C. albicans* are not mediated by conventional activators of fungal immunity such as Dectin-1/CARD9 (Bishu et al., 2014) but predominantly by EGFR (Ho et al., 2019). A model of how candidalysin activates host immunity is presented in Figure 2.

The role of candidalysin during *C. albicans* gut infections is unclear. *In vitro* data indicate that *C. albicans* may translocate across the gut barrier using a combination of processes, including active penetration and cellular damage that may lead to loss of epithelial integrity (Allert et al., 2018). The current view is that candidalysin-induced epithelial damage is only required for *C. albicans* translocation *via* the transcellular route but not the paracellular route. *In vivo* studies are required to fully determine the role of candidalysin in gut infections.

### Macrophages, Dendritic Cells, and Candidalysin

*Candida albicans* forms hyphae once phagocytosed by macrophages, which results in inflammasome activation, cell lysis, and escape. Inflammasome activation requires a priming step and an inflammasome-activating step (Latz et al., 2013). In mouse and human primary monocyte-derived macrophages and dendritic cells, candidalysin (5–50 μM) was able to provide the signal to activate the NLRP3 inflammasome, resulting in caspase-1-dependent maturation and secretion of IL-1β (Kasper et al., 2018; Rogiers et al., 2019). As such, candidalysin was shown to promote systemic infection in an intravenous murine model of disseminated candidiasis (Swidergall et al., 2019).

### Microglial Cells and Candidalysin

Within the central nervous system, the C-type lectin receptor/Syk adaptor CARD9 facilitates protective antifungal immunity through neutrophil recruitment (Drummond et al., 2015). Candidalysin (3–20 μM) was recently shown to induce chemokine (C-X-C motif) ligand-1 (CXCL1) and IL-1β secretion from CARD9^+^ microglial cells in a p38/c-Fos dependent manner, which recruit CXCR2-expressing neutrophils to the brain to control *C. albicans* infection (Drummond et al., 2019).

## Protein Toxins: Mucoricin

Mucormycosis is an often lethal infection in immunocompromised individuals caused by fungi in the order mucorales. Recent research has identified a 17 kDa ricin-like protein toxin called mucoricin [encoded by *RLT1* (Ricin-Like Toxin-1)] which plays a critical role in the pathogenicity of *Rhizopus delemar* hyphae (Soliman et al., 2021). Treatment of primary alveolar epithelial cells with 29.4 μM mucoricin *in vitro* caused significant cellular damage after 3 h, while mice that received an intravenous inoclulation of 5.9 μM mucoricin every second day exhibited a 65% reduction in survival after 5 days compared to controls (Soliman et al., 2021). Histological examination of organs collected from tretaed mice revealed necrosis, haemorrhage and inflitration of immune cells in lung and liver tissue. Notably, RNAi knockdown of mucoricin expression was observed to attenuate the pathogenicity of *Rhizopus delemar in vivo*. Mucoricin uses N-glycosylase activity to inhibit protein synthesis, a process which is followed by RNA depurination (Soliman et al., 2021). Intruigingly, orthologues of *RLT1* have been identified in other fungi and bacteria (Soliman et al., 2021), suggesting that mucoricin-like proteins may be more widespread in nature. The modulation of host immune responses by mucoricin is summarised in Figure 2.

## Conclusion

The biological impact of mycotoxins, peptide and protein toxins on health is significant. Gliotoxin, aflatoxins and candidalysin have been detected in patients with active fungal infections and in experimental models of disease. Furthermore, toxins which are not associated with active infection *per se* can nevertheless be acquired by ingestion, which can cause acute toxicosis. A common theme that emerges from experimental investigations is the breakdown of mucosal barriers, induction of cell death and the suppression of immune cell function in response to toxin exposure, which may increase host susceptibility to microbial infection. The numerous influences exerted by fungal toxins on host immune defence are summarised in Table 1. While the majority of *in vitro* mycotoxin studies and those performed in animal models *in vivo* use a range of toxin concentrations in which specific biological responses are observed, more work is required to establish the relevance of such concentration ranges in the context of acute toxicity and low-level chronic exposure in humans. Further research will undoubtedly increase our understanding of the influences exerted by fungal toxins on health and disease.

**TABLE 1:** Modulation of host defences by mycotoxins, peptide, and protein toxins.

**Toxin**	**Function**	**References**
Gliotoxin	• Induces apoptosis (macrophages/dendritic cells/monocytes/eosinophils) • Inhibits phagocytic function (macrophages/neutrophils) • Induces ROS production (macrophages) • Cytoskeletal remodelling (macrophages) • Inhibits formation of lamellipodia (macrophages) • Inhibits cytokine production (macrophages/dendritic cells/monocytes/eosinophils) • Inhibits activation (neutrophils) • Inhibits filopodia production (neutrophils) • Induces F-actin reorganisation (neutrophils) • Reduces ROS production (neutrophils) • Inhibits NADPH oxidative burst (neutrophils)	•Mullbacher and Eichner, 1984 •Waring et al., 1988 •Suen et al., 2001 •Levin et al., 2015 •Schlam et al., 2016 •Orciuolo et al., 2007 •Coméra et al., 2007 •Tsunawaki et al., 2004 •Stanzani et al., 2005 •Fujihara et al., 2002 •Johannessen et al., 2005

Aflatoxins	• Inhibits phagocytosis (macrophages) • Inhibits NO production (macrophages) • Induces cell cycle arrest (macrophages) • Induces formation of METs • Inhibits intracellular ROS (high dose: neutrophils) • Induces intracellular ROS (macrophages/neutrophils) • Inhibits chemotactic responses (neutrophils) • Inhibits intracellular killing of pathogens (neutrophils) • Induces cytokine expression (2 h: dendritic cells) • Reduces phagocytic ability (12 h: dendritic cells) • Inhibits T-cell activation (12 h: dendritic cells) • Reduces TEER (epithelial cells) • Reduces expression of tight junction proteins (epithelial cells) • Increases paracellular permeability (epithelial cells)	•Stanzani et al., 2005 •Orciuolo et al., 2007 •Bianco et al., 2012 •An et al., 2017 •Mehrzad et al., 2020 •Ubagai et al., 2008 •Mehrzad et al., 2011 •Mohammadi et al., 2014 •Mehrzad et al., 2014 •Caloni et al., 2012 •Gao et al., 2017

Ochratoxin	• Inhibits IL-1α/TNF-α production *in vivo* (macrophages) • Cell cycle arrest and induction of apoptotic genes (macrophages) • Induces IL-1β, IL-6 and TNF-α *in vitro* (macrophages) • Induces necrosis (neutrophils) • Reduces NO production (neutrophils) • Reduces chemotactic responses (macrophages/neutrophils) • Reduces phagocytic responses (neutrophils) • Reduces TEER (epithelial cells) • Inhibits expression of tight junction proteins (epithelial cells)	•Dhuley, 1997 •Brennan et al., 2017 •Müller et al., 1995 •Kupski et al., 2016 •Richetti et al., 2005 •McLaughlin et al., 2004 •Wang et al., 2017

Fumonisin	• Induces apoptosis (macrophages) • Inhibits phagocytic ability (macrophages) • Inhibits expression and secretion of cytokines (macrophages) • Inhibits maturation (dendritic cells)	•Grenier et al., 2011 •Qureshi and Hagler, 1992 •Liu et al., 2002 •Devriendt et al., 2010

T-2	• Induces apoptosis (macrophages) • Inhibits NO production (macrophages) • Inhibits oxidative burst (macrophages) • Inhibits secretion of inflammatory cytokines (macrophages) • Induces formation of balloon-like protrusions (BLPs: mice) • Induces cell death (dendritic cells/monocytes) • Inhibits maturation and activation (dendritic cells) • Inhibits differentiation (monocytes)	•Seeboth et al., 2012 •Hymery et al., 2009 •Chikina et al., 2020 •Hymery et al., 2006

Deoxynivalenol	• Induces release of cytokines (macrophages/neutrophils/epithelial cells) • Induces apoptosis (macrophages/neutrophils) • Reduces chemotactic response (neutrophils) • Reduces TEER and tight junction protein expression (epithelial cells) • Induces Th17 immune response (epithelial cells) • Induces colitis-like symptoms in rats	•He et al., 2013 •Moon and Pestka, 2002 •Chung et al., 2003 •Islam et al., 2006 •Marzocco et al., 2009 •Sugiyama et al., 2016 •Gauthier et al., 2013 •Pinton et al., 2010 •Cano et al., 2013 •Payros et al., 2020

Patulin	• Inhibits expression and secretion of cytokines (macrophages) • Inhibits intracellular killing of fungi (macrophages) • Induces apoptosis (macrophages/epithelial cells) • Inhibits NO production (macrophages) • Induces mitochondrial dysfunction and mitophagy (macrophages) • Reduces TEER (epithelial cells) • Inhibits expression of ZO-1 (epithelial cells) • Inhibits ZO-1 and claudin-4 production (epithelial cells)	•Oh et al., 2015 •Bourdiol et al., 1990 •Tsai et al., 2016 •Luft et al., 2008 •Assunção et al., 2016 •Kawauchiya et al., 2011

Zearalenone	• Induces inflammatory cytokines (macrophages/epithelial cells) • Inhibits protein/DNA synthesis (epithelial cells) • Induces apoptosis (macrophages/epithelial cells) • Activates inflammasome (epithelial cells) • Induces colitis-like symptoms in mice	•Abid-Essefi et al., 2003 •Kouadio et al., 2005 •Pestka and Zhou, 2006 •Fan et al., 2017 •Fan et al., 2018

Candidalysin	• Induces NLRP3 inflammasome activation (macrophages) • Damages plasma membranes (epithelial cells) • Induces release of cytokines (epithelial cells/neutrophils/macrophages) • Induces calcium influx (epithelial cells) • Induces EGFR activation (epithelial cells)	•Kasper et al., 2018 •Verma et al., 2017 •Moyes et al., 2016 •Ho et al., 2019

Mucoricin	• Inhibits protein synthesis (N-glycosylase activity) • Induces hypovolemic shock in mice • Causes apoptosis and necrosis (murine lung and liver tissue)	•Soliman et al., 2021

## Author Contributions

RB wrote the initial draft. EP created figures and figure legends. All authors contributed to manuscript editing.

## Conflict of Interest

The authors declare that the research was conducted in the absence of any commercial or financial relationships that could be construed as a potential conflict of interest.
